# Fluorescent Nitrogen-Doped Carbon Dots for Label Live Elder Blood-Stage *Plasmodium falciparum* through New Permeability Pathways

**DOI:** 10.3390/molecules27134163

**Published:** 2022-06-29

**Authors:** Jiahui Xu, Fengyue Hu, Shuang Li, Jiaojiao Bao, Yi Yin, Zhenyu Ren, Ying Deng, Fang Tian, Guangyu Bao, Jian Liu, Yinyue Li, Xinlong He, Juqun Xi, Feng Lu

**Affiliations:** 1 Jiangsu Key Laboratory of Experimental and Translational Non-Coding RNA Research, School of Medicine, Yangzhou University, Yangzhou 225009, China; xujiahui901@163.com (J.X.); hfy941218@163.com (F.H.); shuangli611@163.com (S.L.); yinyi523@163.com (Y.Y.); renzhenyv@163.com (Z.R.); 19895328720@163.com (Y.D.); fangtianf@163.com (F.T.); lyy1242602847@163.com (Y.L.); hexl@yzu.edu.cn (X.H.); 2 Affiliated Hospital of Yangzhou University, Yangzhou 225000, China; Joe_mine@163.com (J.B.); baogy2004@163.com (G.B.); yzyysxk@126.com (J.L.); 3 The Third People’s Hospital of Yangzhou, Yangzhou 225012, China; 4 Jiangsu Key Laboratory of Zoonosis, Jiangsu Co-Innovation Center for Prevention and Control of Important Animal Infectious Diseases and Zoonoses, Yangzhou 225009, China

**Keywords:** *Plasmodium falciparum*, nitrogen-doped carbon dots, new permeability pathways, flow cytometry

## Abstract

To verify the size and emergence time of new permeability pathways (NPPs) in malaria parasites, the permeability of the *Plasmodium falciparum*-infected erythrocytes was tested with different particle sizes of nanomaterials by flow cytometry assay. The results confirmed the permeability of the host cell membrane increases with parasite maturation for the stage-development evolution of NPPs, and especially found that a particle size of about 50 nm had higher efficiency. As a kind of the novel nanomaterials, nitrogen-doped carbon dots (NCDs) showed no toxicity, specificity binding ability to the malaria parasites, and could label live elder blood-stage *P. falciparum* through NPPs, indicating the potential application in cell imaging. NPPs and some nanomaterials such as NCDs deserve more attention and exploration for the elimination and prevention of malaria.

## 1. Introduction

Malaria caused by the apicomplexan protozoan of the *Plasmodium* genus, remains one of the most prevalent parasitic diseases in the world. There were an estimated 14 million more malaria cases and 47,000 more deaths in 2020 compared to 2019, due to disruption of services during the pandemic [1]. In the human hosts, erythrocyte stages of malaria parasites were the main period cause symptoms [2,3]. To survive within erythrocytes, malaria parasite causes major changes in the structure, composition, and function of the host plasma membrane [4,5]. For the survival of parasite, it appears that “New permeation pathways” (NPPs) with pore-like properties induced in membranes of infected blood cells (iRBCs), which allow anions, small nonelectrolytes, and also macromolecules gain access to the parasite [6,7,8]. Furthermore, beads with a diameter of less than 80 nm were found to be capable of accessing intracellular parasites in iRBCs [9]. NPPs play an essential role in parasite development, and show stage-dependent evolution [10]. As an important channel to connect the internal parasite and outside, NPPs are obvious excellent routes to explore the internal living parasites.

As technology advances, nanotechnology with interdisciplinary approaches has potentially changed the entire scenario of the biomedicine. Nano refers to a size scale of 100 nm or less, nanoparticles have enormous applications in biomedical field for the unusual properties such as optical, physical, biological, and so on. Fluorescent microspheres were designed with a known diameter which make them ideal reference standards for calibrations, sensitive image registration, and quantitative measurements with stained fluorescent cells. Carbon dots (CDs) with a size of less than 10 nm, tunable fluorescence, and strong fluorescence merit to be used as intracellular imaging probes [11]. Fluorescent carbon dots have emerged as a feasible alternative to the more well-known quantum dot or molecular probe [12,13,14]. The functionality of CDs could be improved by modulating their electronic structure, such as doping with heteroatoms [15]. In particular, nitrogen-doped carbon dots (NCDs), have attracted considerable interest due to their advantageous features, including photoluminescence and photostability [16]. Moreover, NCDs show great advantages in cell penetration and imaging [17], which indicates that NCDs could be potential application in the imaging of live malaria parasites through NPPs.

Bioluminescent *P. falciparum* lines have been frequently employed in parasite biology research as well as experiments requiring the detection and quantification of parasite development or maturation [18,19,20,21]. However, there are significant drawbacks to this approach, such as low transfection efficiency and, as a result, the long time it takes to develop stable transfectants [22,23]. Based on the properties of nanomaterials and malaria parasites, here we attempted to verify NPPs in blood-stage *P. falciparum* with florescent microspheres, and further explore the potential application of NCDs for labeling the internal living parasites.

## 2. Results and Discussion

### 2.1. Synthesis and Purification of NCDs

**Figure 1:**
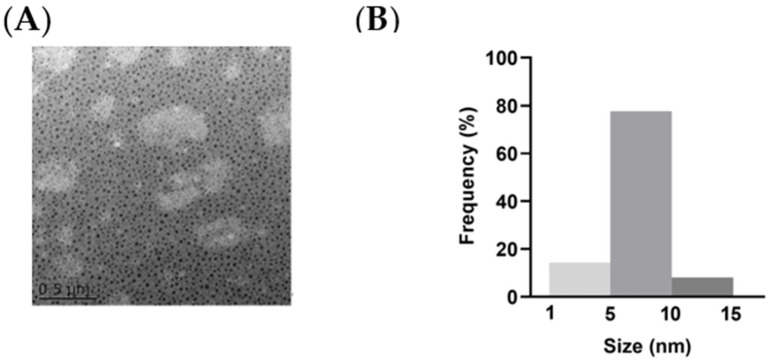
Characterizations of NCDs. (**A**) Morphology of NCDs in TEM measurement. (**B**) Size distribution of NCDs.

We successfully synthesized and purified the NCDs and labeled the NCDs with fluorescein isothiocyanate isomer (FITC). The image of transmission electron microscopy (TEM) showed the spherical nature of the prepared NCDs (Figure 1A). The average size histogram (Figure 1B) revealed that 77.6% of particles were dispersed over the range 5~10 nm with an average size of 8 nm.

### 2.2. Detection of P. falciparum-Infected Erythrocytes by Flow Cytometry with TO and HE

The two commonly used nucleic acid dyes, Thiazole Orange (TO) and Hydroethidine (HE), were utilized to establish the flow cytometry assay. Comparing with normal RBCs, iRBCs showed clearly defined populations at different stages. Flow cytometry revealed that the majority of the ring-stage parasites had weaker fluorescence, and the peak was higher and narrower (Figure S1(Ab)). In contrast, the fluorescence intensity of the trophozoite and schizont stages gradually increased, and the peak gradually fell and broadened (Figure S1(Ac,d)). No significant difference was observed between TO and HE (Figure S1B). Comparing to the Giemsa-based microscopic analysis, TO/HE-based flow cytometry assays were reliable, indicated about 95% of the parasitemia.

The technology of flow cytometry used in this study helped us work out the results rapidly and correctly which provides the opportunity to get more information about malaria parasites [24]. HE is a nucleic acid fluorochrome which transformed by metabolizing cells to ethidium. After incubating with HE, live and dead leukocytes and iRBCs and RBCs could all be identified based on the fluorescence intensity and size, as a previous study showed [25]. TO is a cyanine stain which is based on thioflavin T and it stains primarily RNA [24]. Both stainings, HE and TO, had high efficiency to detect parasites at the blood stage as described in previous study and were confirmed again in this study [26,27].

### 2.3. Detection of P. falciparum-Infected Erythrocytes by Flow Cytometry with Nanomaterials

**Figure 2:**
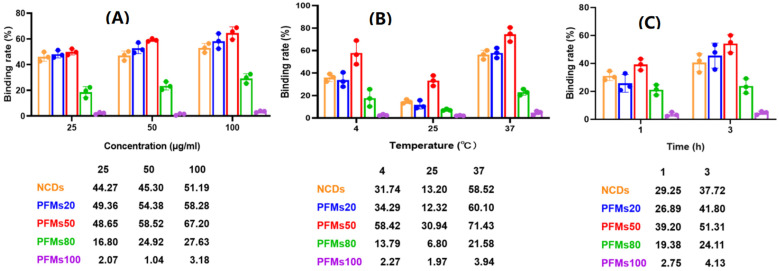
Optimization of permeability of the *P. falciparum*-infected erythrocytes with non-synchronized culture. Three factors are involved, including (**A**) concentration: 25, 50 and 100 µg/mL; (**B**) temperature: 4 °C, 25 °C and 37 °C, (**C**) incubation time: 1 h and 3 h. One factor was tested with the optimal conditions of other two factors. *x*-axis indicates incubation condition, *y*-axis indicates binding rate. The data below this chart showed specific binding rate of nanomaterials.

NCDs and polystyrene fluorescent microspheres (PFMs) with particle size of 20 nm, 50 nm, 80 nm, 100 nm, which were marked as PFMs20, PFMs50, PFMs80, and PFMs100, were serial diluted (25 µg/mL to 100 µg/mL) and incubated with asynchronous iRBCs (6.28% parasitemia, schizont dominated) for 1 h and 3 h at 4 °C, 25 °C, and 37 °C, respectively, compared with TO as a positive control. The optimal incubation conditions were determined by the above experimental operations. As shown in Figure 2 and Figure S2, the largest percentage fluorescent events of NCDs, PFMs20, PFMs50, PFMs80, and PFMs100 were 3.10%, 4.03%, 4.32%, 2.00%, and 0.44%, respectively, and the relative binding rates were 51.19%, 58.28%, 67.20%, 27.63%, and 3.18%, respectively, which were achieved with 100 µg/mL, 3 h and 37 °C. The five fluorescent nanomaterials showed special parasites binding ability except PFMs100, and PFMs80 showed lower ability compared to others. For the binding rate of nanomaterials changed consistently at different incubation temperatures, NCDs and PFMs20 were selected for experimental verification. The results were consistent and indicated the lowest binding rate at 25 °C (Figure S3A,B). The binding efficiency was also different for the incubation time, 3 h was better than 1 h.

**Figure 3:**
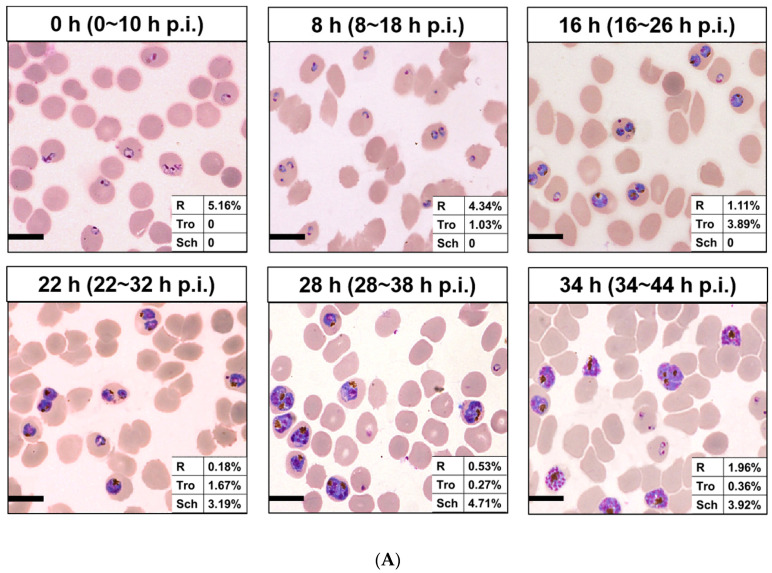

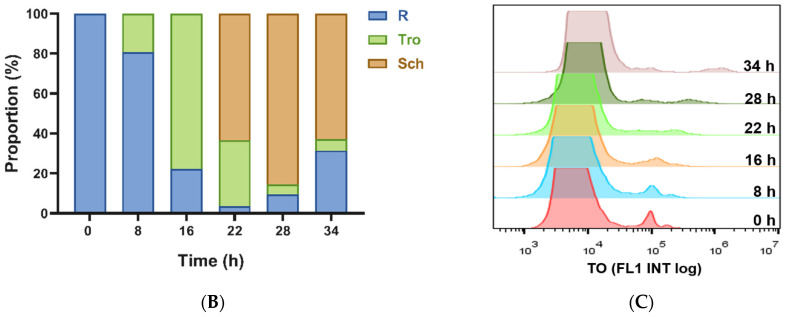
Different interval cultures of *P. falciparum* 3D7 after two consecutive sorbitol treatments (0, 8, 16, 22, 28m and 34 h). (**A**) Giemsa-stained thin blood smears of the culture revealed developmental stages of iRBCs. The stage windows were shown in brackets, indicated the age post infection. (**B**) Proportion of rings, trophozoites and schizont at different time points. (**C**) Overlaid histogram of iRBCs obtained from staining with TO for determining the fluorescence intensity of each stage of iRBCs. All scale bars: 10 µm. R, ring; Tro, trophozoite; Sch, schizont.

**Figure 4:**
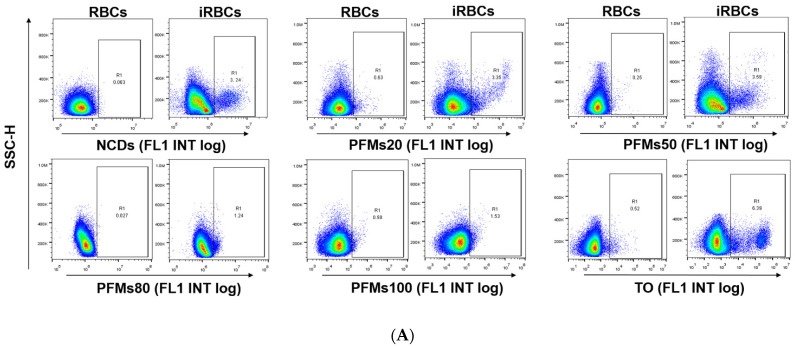

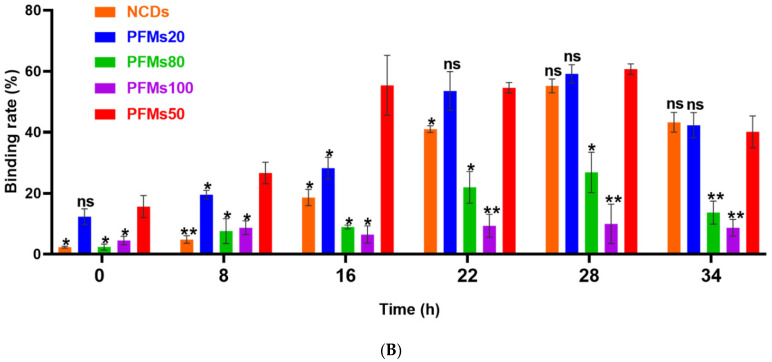
Permeability of *P. falciparum*-infected erythrocytes depends on intra erythrocytic development of Plasmodium and the size of nanomaterials. (**A**) The iRBCs at 28 h after synchronizations were stained with five different nanomaterials, respectively, gating was done according to RBCs. TO as a positive control. (**B**) The binding rates of different size of nanomaterials with different interval cultures. The asterisks denote a significant difference from PFMs50. ** *p* < 0.01, * *p* < 0.05, ns > 0.05, ns: no significance.

Then, 100 µg/mL of each nanomaterial is incubated with different interval cultures after synchronization (0, 8, 16, 22, 28, and 34 h) for 3 h at 37 °C. Parasites were synchronized using two rounds of 5% D-sorbitol treatment to get a stage window of 0~10 h post infection (p.i.) out of the 42~48 h of the cycle. As the result, only ring stage parasites were observed at 0 h after synchronization, 80.7% rings and 19.3% early trophozoites at 8 h, 22.2% rings and 77.8% medium age trophozoites at 16 h, and schizonts occurred at 22 h (63.3%), which reached the highest at 28 h (85.5%), then decreased for partial mature schizonts rupture to enter a new cycle at 34 h (Figure 3). Of all the nanomaterials used here, PFMs20 and PFMs50 showed higher binding efficiency, especially PFMs50, and PFMs100 was the lowest (Table 1, Figure 4). The binding rates were increased with parasites development, low at 0 h, and reached the top at 28 h. At 0 h, only ring stage existed, and the binding rates of NCDs, PFMs20, PFMs50, PFMs80, and PFMs100 were 2.30%, 12.32%, 15.63%, 2.38%, and 4.49%, respectively, compared to 28 h, which were 55.18%, 59.17%, 60.08%, 26.83%, 26.83%, and 9.95%, respectively (Figure S4 and Figure 4, Table 1). When the schizonts became the main stage at 22 and 28 h, the binding rates of the nanomaterials were increased except PFMs100, and the highest binding rate of PFMs80 was only 26.83%. As a positive control, the binding rate of TO was about 95% throughout (Figure 4B, Table 1).

As an anion-selective channel, NPPs are the penetration path of numerous essential nutrients required for the survival of parasites [8], and also fluorescently labeled latex beads with particle size less than 80 nm can also pass through this channel [9]. As expected and shown before, special binding was not observed with PFMs100. However, the binding efficiency was not correlated with the size tightly, even PFMs80 was lower than others with smaller size, PFMs50 was the highest, then PFMs20, and NCDs. Based on our study, the optimal size of materials pass through NPPs is about 50 nm. By analogy, antimalarial nanocarriers with approximately 50 nm in diameter could access furthest intracellular parasites in iRBCs.

The permeability of the host cell membrane increases with parasite maturation for the stage-development evolution of NPPs, reaching a peak at approximately 36 h p.i. [28]. Normally, NPPs generate by parasite activity, and occur 12~16 h after *P. falciparum* invasion [29]. However, NPPs are fully deployed in the majority of iRBCs until the mid-trophozoite stage, and evolve differentially throughout the ring stage [10]. As a kind of carbohydrate, sorbitol can pass through NPPs [30], and is used in the osmotic decomposition method for parasite synchronization. With two consecutive sorbitol treatments, we got a stage window of 0~10 h p.i. Starting from this early ring stage, parasites were cultured and the binding efficiency was checked with fluorescent nanomaterials intervals. Because of incubation for 3 h, the eldest parasites could be 13 h, then weak binding was observed for PFMs20 and PFMs50 at 0 h, and reaches the peak at 28 h (28~38 h p.i.), which was consistent with the results presented previously [6].

### 2.4. NCDs Show No Toxicity to P. falciparum-Infected Erythrocytes

Different from the low viability of parasites in the positive control chloroquine wells, the parasites grew well and no significant difference was seen with the negative control wells (iRBCs only) even with the highest concentration of NCDs (200 µg/mL). The results of SYBR Green I-based drug sensitivity assay were consistent with the microscopic way, indicating no toxicity of NCDs with the tested concentrations.

Accordingly, we confirmed the low cytotoxicity of NCDs to blood stage of malaria parasites, the results showed no toxicity of NCDs to the parasites even at the highest concentration of 200 µg/mL, which provided a basis for further research in malaria field [31].

### 2.5. Determination the Distribution of NCDs in P. falciparum-Infected Erythrocytes

**Figure 5:**
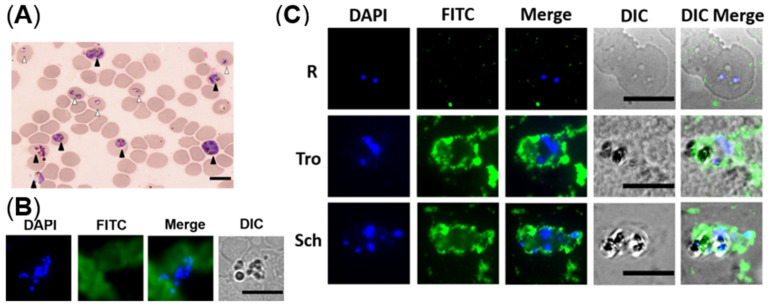
The distribution of NCDs in *P. falciparum*-infected erythrocytes. (**A**) Giemsa-stained thin blood smear of the non-synchronized culture revealed mixed developmental stages of iRBCs. White and black triangles indicate ring and schizont, respectively. (**B**) Fluorescent images of fixed iRBCs with NCDs. (**C**) Fluorescent images of live iRBCs at different stages with NCDs. DAPI was used for nuclear staining; NCDs were labeled with FITC; DIC (differential interference contrast): transmission light microscopy images of iRBCs. All scale bars: 10 µm.

After microscopic analysis, the cultured iRBCs with mixed developmental stages were analyzed in two ways. One was following the steps of IFA, blood smears were prepared before incubation with NCDs. It was found that NCDs were all outside of the cells and no special binding of the parasites was seen (Figure 5B). In the contrast, another way was incubating NCDs with the cultured parasites ahead, then making slides and observing by fluorescent microscopy. The results indicated that NCDs could enter the cytoplasm of iRBCs at trophozoite and schizont stages, instead of young ring stage forms (Figure 5C).

Meanwhile, PFMs20 and NCDs were done in parallel with the second method. Different incubation temperatures, 4 °C, 25 °C, and 37 °C were tested simultaneously, no significant differences were observed between the two nanomaterials. Consistent with the flow cytometry assays, the highest binding efficiency was under 37 °C, and the lowest was 25 °C (Figure S3). In terms of incubation temperature, when nanomaterials were incubated with Plasmodium at 25 °C, both flow cytometry and fluorescence microscopic imaging showed the binding rate was significantly reduced. We preliminarily infer that 25 °C will affect the activity of NPPs, but the specific reason needs further experimental verification.

The NCDs that we used were dispersed over the range 5~10 nm with an average size of 8 nm. After NPPs occur, NCDs can penetrate the iRBC with low toxicity and good biocompatibility. Therefore, NCDs could be an ideal fluorescent probe for live elder blood-stage *P. falciparum*. However, it was reported that the displayed excitation-independent behavior of NCDs, and NCDs derived from the hydrothermal synthesis of different amino acids have a variation in the fluorescence [28,32]. In our study, the NCDs were labeled with FITC for detection, then the possibility of own fluorescence application, and how to improve the special binding efficiency are still worth to be further explored.

## 3. Materials and Methods

### 3.1. Sources of Nanomaterials

PFMs contain green fluorescent dyes (Ex 488/Em 525). Particle sizes of 20, 50, and 100 nm fluorescent microspheres were obtained from ACME Microspheres Inc. (Indianapolis, IN, USA), and 80 nm fluorescent microspheres was obtained from Beijing Dk Nano technology Co., LTD. (Beijing, China).

The NCDs aqueous solution was prepared according to the literature with some modifications [33]. In details, 5 mM ammonium citrate was dissolved into 10 mL water and 335 mL ethylenediamine was added. Then the solution was transferred into 25 mL Teflon-lined autoclave, heated at 200 °C for 5 h. After the solution was cooled down to room temperature, the obtained product was subjected to dialysis (MWCO of 3500 Da) for 24 h to acquire the final NCDs aqueous solution. Partial of the synthesized NCDs were conjugated with FITC (Sigma, St. Louis, MO, USA) using the method as described [34] with minor modification: FITC (20 µL, 50 mg/mL) was added to the NCDs (200 µL, 5 mg/mL) solution, adjusted the pH to 8.5 with the total volume 1 mL, stirred for 12 h at 4 °C under dark. At last, the NCDs-FITC conjugates were dialyzed against phosphate-buffered saline (PBS) overnight to remove the unreacted dye.

### 3.2. Parasite Cultivation and Synchronization

*Plasmodium falciparum* strain 3D7 was obtained from Malaria Research and Reference Reagent Resource Center, and routine cultured in vitro as previously described [35]. Briefly, *P. falciparum* 3D7 was cultured in human erythrocytes by standard methods under a low oxygen atmosphere at 37 °C. The complete medium was RPMI 1640 supplemented with gentamicin, NaHCO_3_, HEPES, AlbuMAX II, and hypoxanthine (Gibco, Carlsbad, CA, USA). The cultures were synchronized by treatment with 5% D-sorbitol (Sigma, St. Louis, MO, USA) twice continuously with 40 h interval when required [36,37]. The blood smears were prepared, fixed with methanol, and stained with Giemsa. The parasitemia of each stage was determined by counting intact iRBCs [38].

### 3.3. Flow Cytometry Analysis

Flow cytometry analysis was carried out as described previously [25]. TO (AAT Bioquest, Sunnyvale, CA, USA) and HE (AAT Bioquest, Sunnyvale, CA, USA) were selected as reference. Briefly, TO was diluted 10,000 times and HE was diluted 200 times with PBS; 100 µL of each diluted dye was mixed with the 5 µL packed cells, and incubated in the dark for 20 min at 37 °C.

For optimal incubation conditions, iRBCs were suspended in diluted concentrations of nanomaterials (25, 50, 100 µg/mL) in PBS and kept in the dark at 4 °C, 25 °C, and 37 °C for 1 h and 3 h, respectively. Then, under the optimal concentration and temperature incubation condition, NCDs, PFMs20, PFMs50, PFMs80, and PFMs100 were incubated with different interval cultures after two synchronizations (0, 8, 16, 22, 28, and 34 h). The binding efficiencies were detected by flow cytometry. All the flow cytometry data acquisition and analysis were performed using a FACS Calibur. The detectors of forward scatter, side scatter, FL1 (for detection of TO and FITC), and FL2 (for detection of HE) were set in logarithmic mode.

### 3.4. SYBR Green I-Based Drug Sensitivity Assay

*In vitro* activity against *P. falciparum* was explored by means of SYBR Green I fluorescence assay which were described by Neils and colleagues [39]. The routine cultured iRBCs were synchronized with 5% sorbitol, the serial diluted NCDs (2000 µg /mL to 200 µg/mL) and the control chloroquine were added to the synchronized ring-stage parasite suspension, to obtain a 2% hematocrit and 0.5–1% parasitemia in triplicate wells of a 96-well plate. After 72 h incubation at 37 °C, the effectiveness was measured by the SYBR Green I-based fluorescence assay [40] in parallel with the microscopic examination.

### 3.5. Fluorescence Microscopic Imaging

Mature parasites were fixed and stained by two different methods to redefine the way in which NCDs enter iRBCs. One is following the step of immunofluorescence assay (IFA) with the blood smears of iRBCs as described [41]. After blocking with 5% skimmed milk in PBS, the slides were incubated with FITC-labeled NCDs and DAPI (Invitrogen, Carlsbad, CA, USA). Images were captured using a Nikon Eclipse 80i Upright Fluorescence Microscope.

Another one is living iRBCs were incubated in PBS with the presence of NCDs and DAPI (1 µg/mL, Invitrogen). After incubation, blood smears were prepared and fixed with glacial acetone for 3 min, images were captured as above.

### 3.6. Statistical Analysis

Statistical analyses were performed using GraphPad Prism (version 8.0; GraphPad, San Diego, CA, USA) and SPSS software (version 16.0; SPSS, Chicago, IL, USA). The experiments were performed in triplicate, and Student’s *t*-test was used to analyze the significance of the different levels between two groups. The data are expressed as the mean ± standard deviation (SD) and a difference with *p* < 0.05 was considered statistically significant.

## 4. Conclusions

In summary, with the flow cytometry assay, we verified the size and emergence time of NPPs, and meanwhile compared the efficiency of nanomaterials with different particle sizes to iRBCs. As one kind of the novel nanomaterials, NCDs showed no toxicity and the specific binding ability to the malaria parasites indicates wide application in the field, such as fluorescent probe, but the application in this field remains to be explored. Some nanomaterials such as NCDs and new permeation pathway (NPPs) deserve more attention and exploration for the elimination and prevention of malaria.

## Figures and Tables

**Table 1:** The binding rate (%) of different size particles with different interval cultures of *P. falciparum* 3D7 after synchronization.

	0 h	8 h	16 h	22 h	28 h	34 h
NCDs	2.30 ± 0.00	4.84 ± 0.01	18.60 ± 0.03	41.02 ± 0.01	55.18 ± 0.02	43.26 ± 0.03
PFMs20	12.32 ± 0.04	19.53 ± 0.04	28.21 ± 0.10	53.54 ± 0.02	59.17 ± 0.02	42.13 ± 0.05
PFMs50	15.63 ± 0.01	26.64 ± 0.04	55.37 ± 0.01	54.60 ± 0.05	60.08 ± 0.07	40.13 ± 0.04
PFMs80	2.38 ± 0.01	7.61 ± 0.02	8.93 ± 0.03	21.92 ± 0.04	26.83 ± 0.06	13.65 ± 0.03
PFMs100	4.49 ± 0.04	8.67 ± 0.03	6.48 ± 0.02	9.33 ± 0.04	9.95 ± 0.08	8.68 ± 0.04
TO	94.25 ± 0.03	97.34 ± 0.01	95.13 ± 0.04	98.21 ± 0.06	96.34 ± 0.03	95.35 ± 0.04

The binding rate reported as mean ± standard deviation.

## Data Availability

Data is contained within the article.

## Supplementary Materials

The following supporting information can be downloaded at: https://www.mdpi.com/article/10.3390/molecules27134163/s1, Figure S1: Flow cytometry gating for determining the position of each stage and quantification of *P. falciparum*-infected erythrocytes. Figure S2: Flow cytometric profiles of iRBCs stained with five nanomaterials at 25, 50, 100 µg/mL, respectively, tested with the optimal temperature and incubation time. Figure S3: Analysis the effect of temperature on *P. falciparum*-infected erythrocytes permeability. Figure S4: Flow cytometry results of nanomaterials with different particle sizes.

## Sample Availability

Samples of the compounds are available from the authors.
