# Supplementary material for: Fluorescent Nitrogen-Doped Carbon Dots for Label Live Elder Blood-Stage Plasmodium falciparum through New Permeability Pathways
Source: Molecules. 2022 Jun 29;27(13):4163. doi: 10.3390/molecules27134163 (PMC9267939; doi:10.3390/molecules27134163)
Supplement: Supplementary file 1 [file molecules-27-04163-s001.zip › Supplementary Materials.pdf]

Supplementary Materials

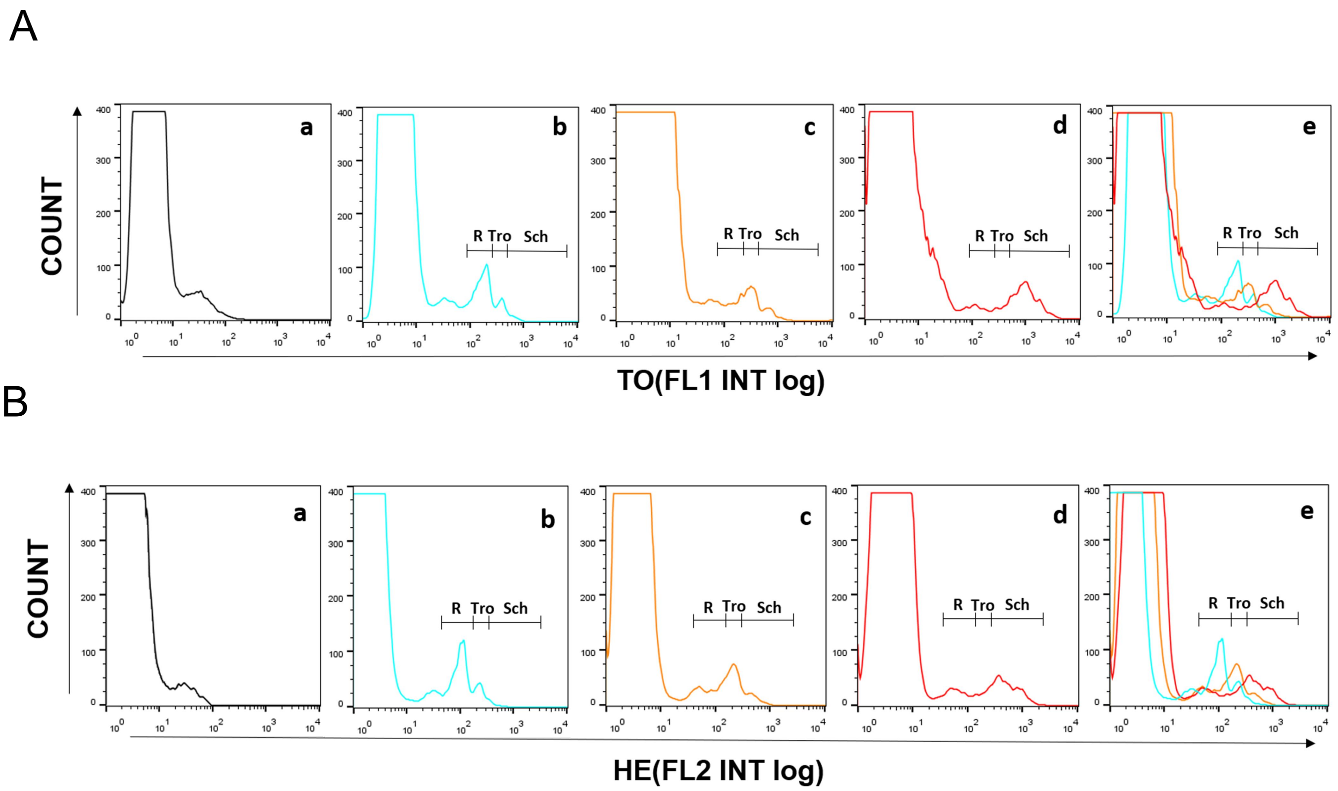

Figure S1. Flow cytometry gating for determining the position of each stage and quantification of *P. falciparum*-infected erythrocytes. Cells were stained with TO (A) and HE (B). The histograms showed that (a) non-infected erythrocytes; (b) ring stage predominant culture; (c) trophozoite predominant culture and (d) schizont predominant culture. (e) Superimposition of histograms from different stages of iRBCs confirmed that TO/HE is able to separate ring stage (R), trophozoite (Tro) and schizont (Sch) and confirmed the validity of the gates.

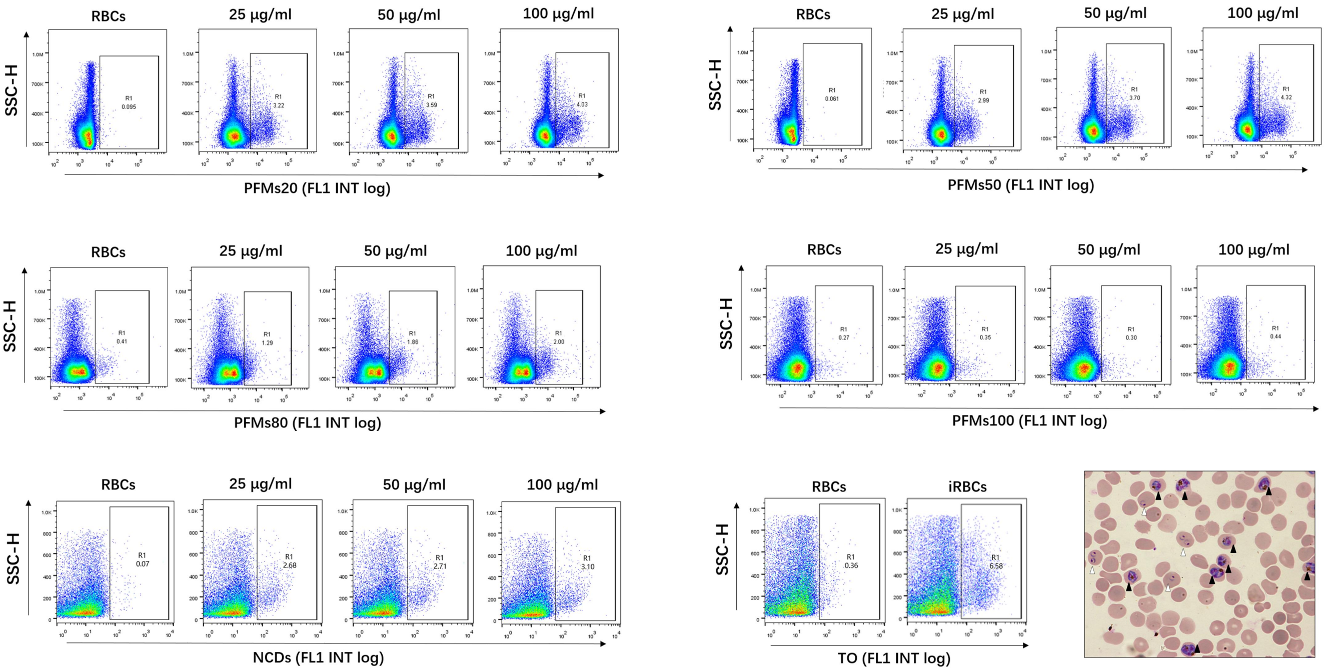

Figure S2. Flow cytometric profiles of iRBCs stained with five nanomaterials at 25, 50, 100 μg/ml, respectively, tested with the optimal temperature and incubation time. Gating was done according to RBCs, TO as a positive control. Giemsa-stained thin blood smear of the non-synchronized culture is shown at the lower right.

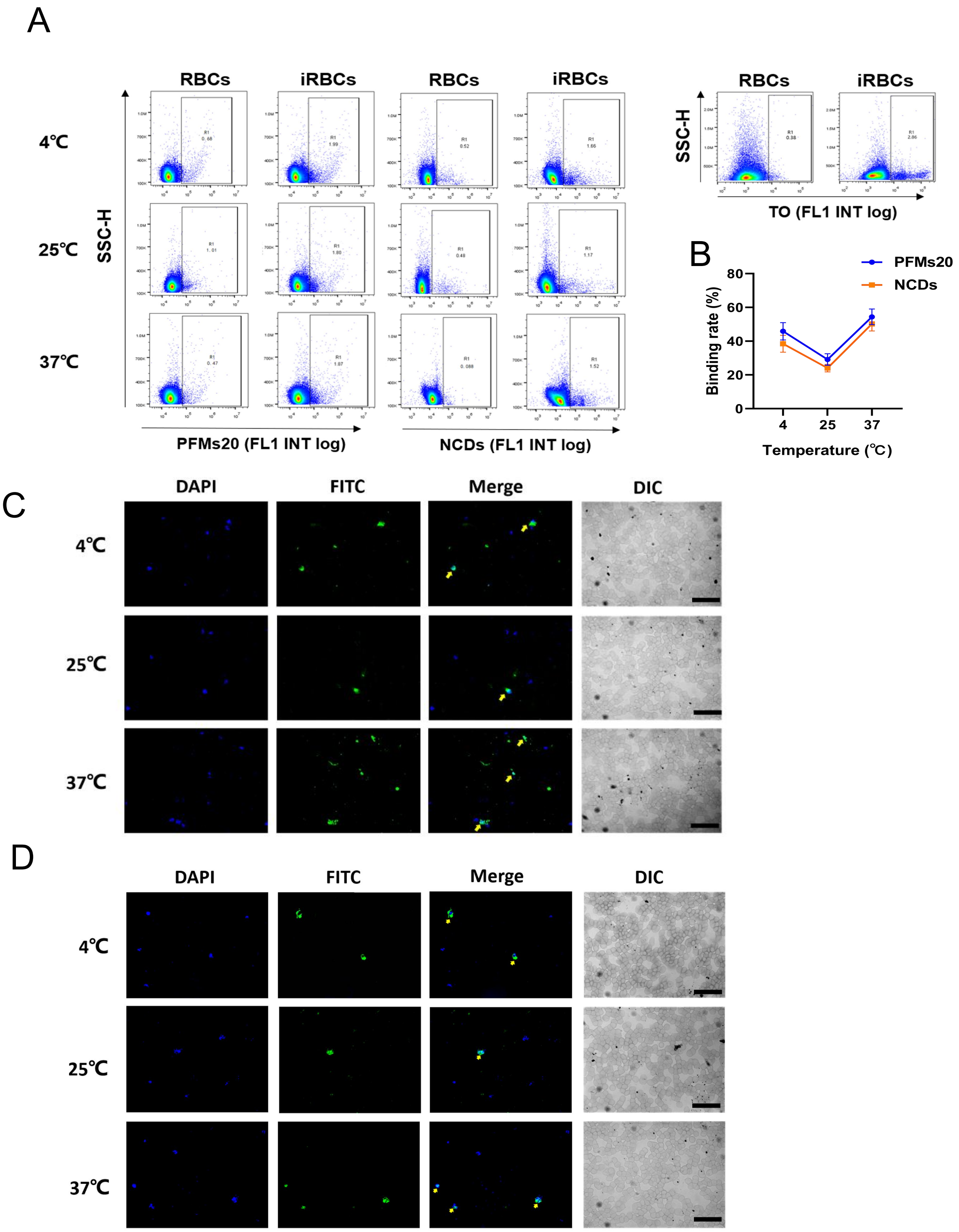

Figure S3. Analysis the effect of temperature on *P. falciparum*-infected erythrocytes permeability. (A) The iRBCs were incubated with optimum concentration of PFMs20 and NCDs at 4°C, 25°C and 37°C, respectively, gating was done according to RBCs, (B) showed the binding rate of PFMs20 and NCDs. (C, D) represented fluorescent images of live iRBCs at different temperature with PFMs20 and NCDs, respectively. DAPI was used for nuclear staining; NCDs were labeled with FITC; DIC (differential interference contrast): transmission light microscopy images of iRBCs. All scale bars: 50 μm.

A

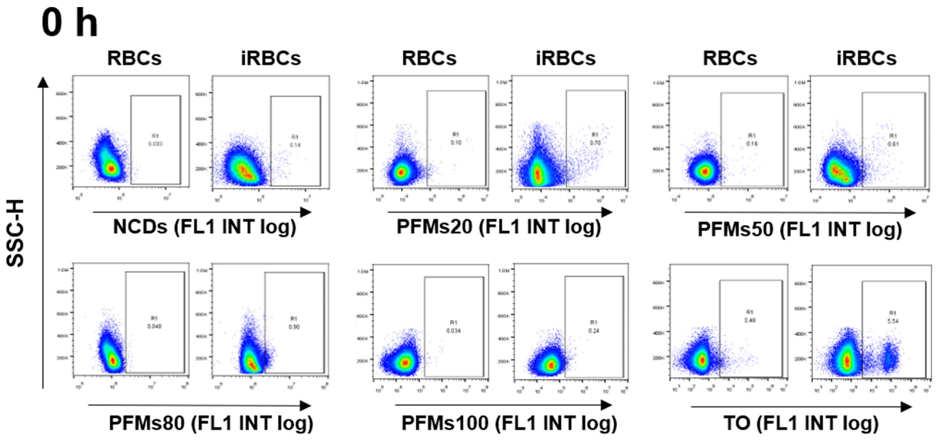
0 h
RBCs
iRBCs
RBCs
iRBCs
RBCs
iRBCs
SSC-H
NCDs (FL1 INT log)
PFMs20 (FL1 INT log)
PFMs50 (FL1 INT log)
PFMs80 (FL1 INT log)
PFMs100 (FL1 INT log)
TO (FL1 INT log)

B

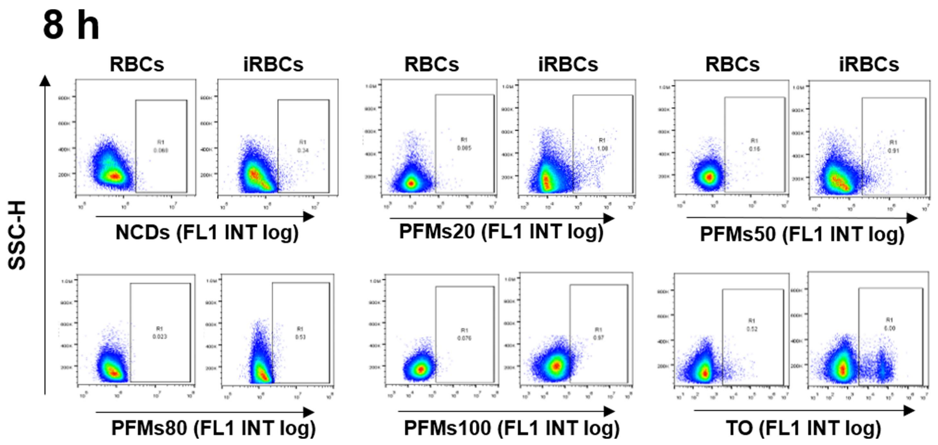
8 h
RBCs
iRBCs
RBCs
iRBCs
RBCs
iRBCs
SSC-H
NCDs (FL1 INT log)
PFMs20 (FL1 INT log)
PFMs50 (FL1 INT log)
PFMs80 (FL1 INT log)
PFMs100 (FL1 INT log)
TO (FL1 INT log)

C

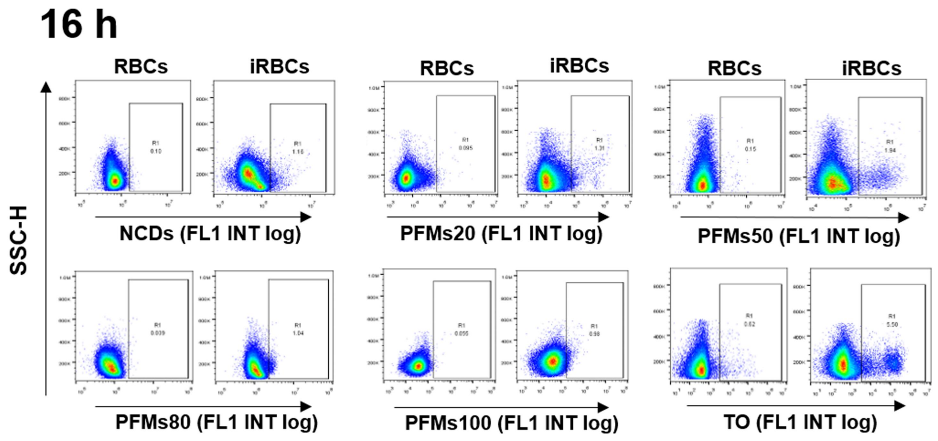
16 h
RBCs
iRBCs
RBCs
iRBCs
RBCs
iRBCs
SSC-H
NCDs (FL1 INT log)
PFMs20 (FL1 INT log)
PFMs50 (FL1 INT log)
PFMs80 (FL1 INT log)
PFMs100 (FL1 INT log)
TO (FL1 INT log)

D

22 h

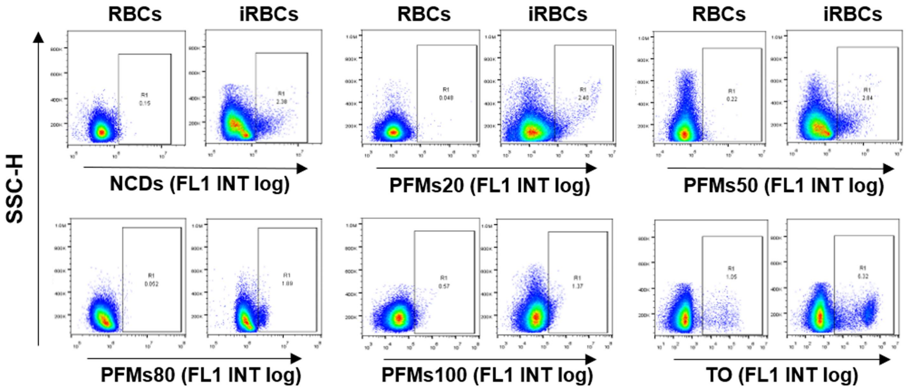

E

34 h

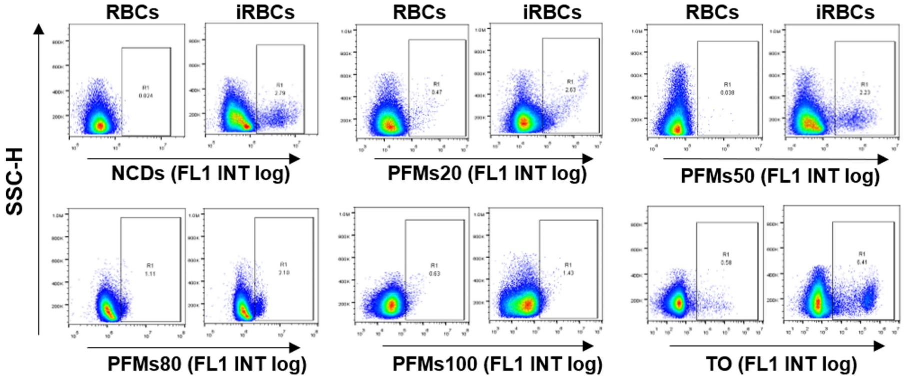

Figure S4. Flow cytometry results of nanomaterials with different particle sizes. (A-E) representative flow cytometry results of NCDs, PFMs20, PFMs50, PFMs80 and PFMs100 at 0, 8, 16, 22, 34 h after two synchronizations, TO as a positive control.
